# Love What You Do and Do What You Love

**DOI:** 10.3389/fped.2015.00119

**Published:** 2016-02-16

**Authors:** Jason Edward Vargas

**Affiliations:** ^1^Pediatrics, Phoenix Children’s Hospital, Phoenix, AZ, USA

**Keywords:** work–life balance, balance, burnout, resident burnout, job satisfaction

From the first day of medical school, the faculty preached an ideal concept of “balance.” It was delivered with the sympathetic belief that it would prepare my classmates and me for a field where a large amount of time would be spent working and studying. More importantly, preparing us for a field where suicide rates have been reported to be higher than the general population (1). It was during our first lecture on balance, unbeknownst to me, that we were all being set up for failure. The concept of balancing “work” and “life” was presented to us as an effortless task. Ideally, I should be able to balance the amount of time I spend doing work equally with the amount of time I spend doing something to improve my quality of life. For me, that would entail going for a hike or spending quality time with family and friends. There are two fundamental problems with this concept: first, in practice, it is not that easy; and second, it categorizes “work” to be the opposite of fun. As I graduated from medical school and endured one more speech about balance, I was left with the words, “don’t forget about the things you love to do.” These words, while at the time were considered to be negligible, have impacted my belief system deeply and have helped to shape my concept of balance.

My view of “work–life balance” may be better appreciated after an understanding of my background and current status. I am a pediatric resident with plans of going into the field of Pediatric Critical Care Medicine. I strongly believe in the value of research and education and have several ongoing projects in addition to my residency clinical requirements and responsibilities. I often find myself pressed for time with barely a moment to contemplate what is work and what is life, never mind balancing the two. Despite all these, I have rarely felt the effects of physical and emotional burnout during my Critical Care rotations, or during residency as a whole. My co-residents do not necessarily share this sentiment. Goldhagen et al. mentions that elevated stress levels lead to burnout, and West et al. states that stress and fatigue lead to increased medical errors (2, 3). As I take a step back, it should not be a surprise that residents fear the field of Critical Care. The shared opinion among most residents is that their Critical Care rotation tends to be the most emotionally and physically trying rotation of their 3 years. As one resident bluntly said, “I avoid that wing like I would the plague.”

It became apparent that the critical care rotation evoked deep seeded emotions for many of my colleagues. In order to determine the possible etiologies, I decided to send out an informal, open-ended survey to each resident. I received an overwhelming number of responses to consider. One resident insightfully wrote:

I feel like I’m a pretty tough person and don’t show much emotion at work, but each time I’ve been in the PICU, I’ve left with a sense of emotional (and physical) exhaustion! You see so much there and some of it is pretty sad. ICU is inherently a place of major burnout, and a winter/busy month can especially feel like that. Further, I think it would be absolutely wonderful if on the final day – maybe over the noon hour, we could all meet with a couple of attendings/NPs, an RN, chaplain or whatever ancillary staff would want to be there to just introduce this concept of burnout and patient loss. We could have an open discussion in how different people deal/cope with what they see after they leave work.

In response to similar sentiments, an intensivist and I instituted a “debriefing session,” at the end of each resident’s monthly Critical Care rotation. This allotted time to discuss the topics surrounding resident stress and burnout. The sessions were set up similar to the system created by Eckleberry-Hunt et al., and Rabow and McPhee in their studies of resident burnout (4, 5). During these sessions, the residents are granted an hour, free of duties, to discuss the rotation, feelings of being overwhelmed, sadness, joy, and their personal methods to avoid burnout. Further emotional services, through social work and the Employee Assistance Program are offered and avenues to connect with them are also provided. These meetings typically are filled with emotion, which is a positive sign that the residents feel comfortable expressing their feelings openly without any omissions. There are two topics that are consistently mentioned at each meeting. Some residents mention a lack of general respect among colleagues. The other is the issue of balancing “work” with “life.”

One resident raised the issue that we all enjoy practicing the art of medicine. However, it is not uncommon to collide with obstacles that obstruct the potential joy that should be found on a daily basis. Attending physicians, nurse practitioners, fellows, co-residents, nurses, and families typically provide the perceived obstacles encountered in medicine. Studies have evaluated the effect of supervisor’s moods on employees and co-workers moods toward each other. Not surprisingly, they found expressed negative emotions toward each other decreased job satisfaction and further initiated negative mood among staff (6, 7).

That being said, it is not uncommon for a co-worker to have a bad day. This is usually due to an unfortunate event or the high stress level, which sadly is part of the job. When this is mixed with poor coping strategies, it can lead to an outward production of negative emotions and cripple another person’s happiness. Thus, giving into the adage, “misery loves company” as seen in the mood influence theory of Bolger et al. and Avramova and Staple. Negative mood reduces cooperation and induces interpersonal conflict (8, 9). There are many potential variables encountered daily that can shatter previous sentiments of adoration toward medicine, which make it just as tough to find enjoyment. This is why it is stressed at each post Critical Care debriefing that we are each other’s support system. We are all responsible for each other continuing to enjoy what we do. Therefore, we should ensure that the opportunity for the emotional and physical balance of our peers exists. No matter what our position is in the hospital, we must realize the impact our attitude plays on our peers and how it can impede the balance required for our success in medicine. More importantly, we must willingly accept this responsibility. The unwillingness to accept this responsibility impacts both life and work balance.

It is remarkable to see many of my colleagues spend their days stressed in the critical care unit and then try to rush home after to do something enjoyable. Not surprisingly, they are disheartened when they realize there is rarely enough time. Their feelings of exhaustion are further amplified when they realize their ideal of work–life balance is unfeasible. This is juxtaposed by others who enjoyed the time spent on their critical care rotation. They did not find themselves falling into a cycle of despondency when they could not get home in time to enjoy the things that bring them pleasure outside of work. Is the latter group more mentally balanced than the former and therefore able to cope with the struggles found in the Critical Care environment? Not necessarily. In fact, it is my contention that the former group has fallen into the unintentional deception of having an equal balance between work and life. Essentially, they assume having a dichotomy will lead to happiness.

By avoiding the dichotomy of work and life as two separate entities, leading to the unintentional assumption of life being good and therefore work being bad, a physician can find balance. So, how does a person avoid creating a system of good and bad that leads to constant disappointment? Is this even possible? Absolutely. I think about the sincere words of my medical school professor urging me not to forget to do what I love. If I live by this belief, I will be a physician who has developed mental and emotional balance. While I try to take time to go for a hike or run, spend time with family and friends, I am realistic that this is not always possible. Establishing myself in a career where free time is fleeting I have to remember that I am doing what I love. Working with patients, figuring out diagnoses, and applying concepts of physiology evokes my inner nerd and brings me an exuberance that I would not be able to experience in any other field. On the days when I find myself working late and cannot meet my friends, I still walk away with a sense of joy and accomplishment. Instead of falling ill to the idea of “work” and “life” needing to be balanced to create harmony, I subscribe to the sentiment that work and life should be one; the struggle to create a dichotomy does the opposite.

Obviously, the issues of work–life balance, job satisfaction, and burnout are multifactorial and hopefully, I was able to reveal a couple of the constituents that play pivotal roles. We need to abandon the outdated belief that creating a dichotomy between work and life will lead to stronger mental and physical balance, and we must accept the responsibility that we all play a vital role in the work–life balance of our colleagues through the expression of our negative and positive attitudes. Each debriefing session that I attend allows me time to reflect on these principles and ensure that I continue to maintain balance in my life, which is essential for a person entering a field that is reportedly time consuming. Lastly, it is comforting to know that while I may have a lot on my plate and cannot afford as much time as I would like for hiking or meeting up with friends, I am happy. I am abiding by the profound words of my medical school professor and not forgetting about the things I love to do.

## Author Contributions

JV – is a Pediatric Resident at Phoenix Children’s Hospital and has a strong interest in Pediatric Critical Care and work–life balance. He has contributed a piece discussing this topic with a strong focus on the obstacles that impede balance in residency.

## Conflict of Interest Statement

The author declares that the research was conducted in the absence of any commercial or financial relationships that could be construed as a potential conflict of interest.
